# Dietary Behavior and Determinants of Diet Quality among Primary Health Care Patients in Poland

**DOI:** 10.3390/nu16070925

**Published:** 2024-03-23

**Authors:** Małgorzata Znyk, Filip Raciborski, Dorota Kaleta

**Affiliations:** 1 Department of Hygiene and Epidemiology, Faculty of Health Sciences, Medical University of Lodz, Żeligowskiego 7/9, 90-647 Lodz, Poland; dorota.kaleta@umed.lodz.pl; 2 Department of Environmental Hazard Prevention, Allergology and Immunology, Warsaw Medical University, Banacha 1a, 02-091 Warsaw, Poland; filip.raciborski@wum.edu.pl

**Keywords:** diet, Mediterranean diet, chronic diseases, DQS, patient, family doctor

## Abstract

Background: The aim of the present research was to determine the factors influencing the prevalence of eating behaviors, diet quality, and unhealthy eating among primary healthcare patients in Poland. Methods: The cross-sectional study included 896 adult primary care patients in Łódź. The study was conducted from January 2020 to December 2021 among thirty-four primary healthcare facilities. A survey recorded the sociodemographic data of the respondents as well as data regarding their health condition and diet. Results: The majority of the respondents (57.6%) had average dietary habits, while 40% had unhealthy eating habits. The univariable logistic regression found that primary care patients with medium/secondary education had a 1.5 times greater risk of unhealthy eating habits, and those with post-secondary vocational education had a 1.75 times greater risk of unhealthy eating habits than those with higher education (OR = 1.46; 95% CI: 1.08–1.97, *p* ≤ 0.01, and OR = 1.75; 95% CI: 1.04–2.94, *p* ≤ 0.05). The multivariable logistic regression confirmed that the level of education had a significant impact on dietary habits: for medium/secondary education, OR = 1.40; 95% CI: 1.03–1.91 (*p* ≤ 0.01); for post-secondary vocational education, OR = 1.69; 95% CI: 1.0–2.85 (*p* ≤ 0.05). Conclusions: The education level was significantly correlated with the prevalence of unhealthy eating behaviors in the studied population. This factor should be considered in the promotion of healthy eating behaviors and nutritional counseling interventions conducted by family physicians in primary health care.

## 1. Introduction

Rapid urbanization and lifestyle changes, including changes in dietary choices, have contributed to an increase in chronic diseases, which has become a public health problem [1]. Chronic diseases are responsible for high rates of mortality and disability worldwide [2], with 74% of deaths worldwide being caused by NCDs (non-communicable diseases) [3]. Chronic respiratory diseases are responsible for 4,1 million deaths a year, cancer for 9.3 million, and cardiovascular diseases (CVD) for 17.9 million [4]. Chronic diseases not only affect the quality of life and health of the population but also have social and economic consequences [1], constituting the dominant part of healthcare spending [5]. The growing burden of chronic diseases has made managing and preventing them a global priority [4]. 

The main reason for the higher incidence of risk factors for chronic diseases is an unfavorable lifestyle [6,7]. Unhealthy diet, overeating, limited physical activity, sedentary lifestyle, and smoking are key factors in the increase of cardiovascular disease, dementia, and some forms of cancer [6,8,9,10,11,12]. Nutrition and diet also play a crucial role in many chronic gastrointestinal diseases. A diet low in fiber and high in animal protein and fats may change the intestinal microbiome, increasing the risk of intestinal diseases and intestinal inflammation. A diet high in simple refined carbohydrates, dairy products, and saturated fats and low in grains, fiber, and vegetables increases the risk of chronic kidney disease and obesity [13].

Numerous studies have demonstrated that a well-balanced diet that provides adequate amounts of essential nutrients reduces the risk of developing NCDs and prevents malnutrition [4]. A healthy diet based on vegetables and fruits, fish, and small amounts of meat products is associated with a lower incidence of cholesterol problems, high blood pressure, type 2 diabetes, stroke, coronary heart disease, and some cancers [14,15]. Dietary choices can impact mental health and neurological diseases. A diet dominated by high-fat, processed foods, and red meat increases the risk of neurological disorders [16].

Healthy eating can protect against psychosocial maladjustment and anxiety [17] and may slow down the activity and progression of multiple sclerosis [18]. Higher diet quality is associated with lower levels of stress [17]. Conversely, excessive consumption of salt and saturated fat may increase the risk of ischemic stroke and affect brain function. Lower diet quality is associated with a higher risk of ischemic stroke [19]. Healthy eating habits and a healthy lifestyle can have a protective effect against chronic diseases and increase life expectancy [3,20]. Promoting a healthy lifestyle is therefore essential to combating chronic diseases and reducing the financial burden on healthcare systems.

Numerous guidelines and recommendations have been developed to guide healthy eating habits. For example, the WHO (World Health Organization) recommends that energy intake from free sugars should not exceed 10%, with an indication to lower this limit below 5%. These guidelines are intended to reduce the incidence of chronic diseases such as diabetes, metabolic syndrome, cardiovascular disease, and obesity [21]. The guidelines recommend choosing mostly plant-based foods and eating five portions of fruit and vegetables a day [22]. The United Nations Food and Agriculture Organization (FAO) and the World Health Organization recommend eating at least two portions of fruit and three portions of vegetables a day [23]. It is recommended to eat optimally fatty fish at least once a week and limit the consumption of (processed) meat [24].

Current dietary guidelines recommend following a healthy eating pattern throughout life. The element that all dietary recommendations have in common is the high quality of the diet [25]. Healthy eating patterns include diets rich in fruits, vegetables, whole grains, low-fat dairy products, and lean protein. Other features of healthy eating patterns include low sodium, saturated fats, trans fats, and added sugars [26]. Changing towards healthy eating patterns can reduce the current high levels of cardiovascular disease, obesity, diabetes, and cancer [26]. Therefore, the combination of a proper lifestyle and healthy eating habits plays an important role in preventing NCDs [20].

The most commonly recommended diet is the Mediterranean diet (MedDiet), characterized by high consumption of fruits, vegetables, extra virgin olive oil, legumes, cereals, and fish, and low consumption of red meat and animal fats. The Mediterranean diet is also characterized by moderate consumption of eggs, dairy products, and red wine [20,24,27,28,29,30,31]. The diet has been found to benefit life expectancy and lower the risk of chronic diseases and cardiovascular problems due to, inter alia, its antioxidant and anti-inflammatory properties. The high intake of vegetables, fiber, and fruit, combined with a low intake of energy-dense processed foods and fats, is believed to help strengthen the immune system and reduce inflammation [32]. The Mediterranean diet is widely regarded as the optimal diet to maintain good health [33]. Indeed, it has been found to have various beneficial effects on health, primarily in the prevention of cardiovascular diseases [34,35], obesity [36], type 2 diabetes [37], and reducing the incidence of breast cancer [38,39,40].

Certain indicators of diet quality are used to assess and evaluate eating behavior, specific dietary patterns, and confirm whether a certain diet is healthy. Diet quality scores have been linked to indicators of metabolic health and nutritional status [41]. 

A simple tool often used by researchers is the Dietary Quality Score (DQS), which examines the four food components that define a healthy diet: fruits, vegetables, fats (cooking and spreadable), and fish [42]. The total score can be used to confirm whether the diet is unhealthy, healthy, or average [42].

Primary healthcare facilities are a good place to conduct lifestyle education activities. Success in the treatment of non-communicable diseases depends, inter alia, on the ability of the doctor to effectively use the resources at their disposal and on their ability to educate patients [43]. Teaching about the importance of physical activity and diet must begin early in life and then be continually reinforced to ensure successful results [43]. Patients feel more comfortable and may be more open to recommendations from a family doctor than from a specialist [44]. Research suggests that patients are most likely to make lifestyle changes if their GP recommends them [45]. GPs should work in multidisciplinary teams with specialists such as dietitians and exercise specialists to promote the adoption of healthy eating patterns and increased physical activity [46].

No research to date has compared the quality of diet among primary care patients in Poland. Therefore, the aim of the present research was to determine the factors influencing the prevalence of eating behaviors, diet quality, and unhealthy eating among primary healthcare patients in Poland.

## 2. Materials and Methods

### 2.1. Characteristics of the Study Sample

The cross-sectional study included 896 adult primary care patients in Łódź, Poland. The study was conducted from January 2020 to December 2021 among thirty-four primary healthcare facilities that agreed to take part. A detailed description of the methodology is described elsewhere [47]. The inclusion criteria included the following elements: age over 18 years, the use of family doctors in primary health care, and consent to participate in the study. The study received permission from the Ethics Committee of the Medical University of Lodz (number RNN/315/18/KE).

### 2.2. The Survey

The participants completed a survey assessing eating behaviors and the quality of the diet. The survey recorded the sociodemographic data of respondents (age, sex, education, marital status, and professional status) and data on their health condition. Another section included information on the frequency of intake of fat, vegetables, fish, and fruit. The questionnaire has been validated in previous studies [48,49]. The section on nutrition used a verified DQS that had been modified for Polish needs [42].

In accordance with WHO recommendations, the questionnaire contains the four most important dietary ingredients responsible for chronic disease risk: fruits, vegetables, fats, and fish. A detailed description of the methodology using the DQS (Dietary Quality Score) is provided elsewhere [49].

Points were assigned to the four dietary components (Table S1, Supplementary Materials). The fat rating is divided into cooking fats and spreadable fats: butter, spread, and lard result in 0 points, vegetable margarine results in 1 point, and no fat results in 2 points. Fat points are summed (Table S1, Supplementary Materials). A portion of fish less than 200 g per week is worth 1 point, more than 200 g per week is worth 2 points, and no fish is worth 0 points. Consuming 2–5 servings of vegetables a day is worth 1 point, consumption of more than 5 servings of vegetables a day is worth 2 points, and less than 2 servings a week is worth 0 points. Consuming more than 3 portions of fruit a day is worth 2 points, less than 3 servings a week is worth 0 points, and 3 servings a week but not less than 2 a day is worth 1 point. 

A total score of 7–8 points indicates healthy eating habits (eats fish, vegetables, and fruits more often, uses fats for cooking, or spreads less often), while 0–3 points indicates an unhealthy diet (low consumption of vegetables, fruit, and fish). Obtaining 4–6 points indicates an average diet (less frequent consumption of vegetables, fruit, and fish, more frequent use of fats for cooking and spreading) (Table S2, Supplementary Materials).

### 2.3. Statistical Analysis

Statistical calculations were performed using the chi-squared test and univariable and multivariable logistic regression. The study population was divided into three groups: average dietary habits, unhealthy dietary habits, and healthy dietary habits. The groups were compared using an extended Mantel–Haenszel chi-squared test. 

For each indicator, the odds ratio (OR) and confidence interval (95% Cl) were calculated for unhealthy eating behaviors using univariable and multivariable logistic regression analyses. The following variables were used to adjust the models: sex, age, level of education, marital status, professional situation, number of protective diseases, and body mass index. 

Variables that were found to be statistically significant (*p* < 0.05) in the univariable logistic regression analyses were included in the multivariable logistic regression analyses. Ordinal logistic regression was used to determine associations between BMI groups and dietary habits.

Data were analyzed using the IBM SPSS program, v. 29.0.0 (IBM Corp., Armonk, NY, USA) and STATISTICA version 13.3 (StatSoft Poland Inc., Kraków, Poland).

## 3. Results

### 3.1. Socio-Demographic Characteristics of the Study Population

Table 1 presents the socio-demographic characteristics of the study population. Of the 896 primary care patients surveyed, 25.8% were men (*n* = 231) and 74.2% were women (*n* = 665). In addition, 56.9% of respondents had secondary education, 44% were single, and 61.6% were professionally active; 46.9% of primary care patients did not have a history of any chronic disease. Approximately 55.1% of participants had a normal body mass index (<25 kg/m^2^).

### 3.2. Dietary Quality Score Characteristics (DQS) among the Study Participants

Table 2 shows the frequency of consumption of the most important food ingredients (fish, fats, fruits, vegetables). About half of the presented population consumed more than five portions of vegetables per week (49.7%), and over half (67.3%) consumed three pieces/week of fruit and <2 pieces/day. About half (47.3%) ate <200 g of fish each week and often consumed fats for spreading (butter, mixed spreads, lard, 74.9%) and cooking fat (vegetable margarine, oil, 56.9%). Half (women 50.1% and men 48.5%) indicated eating more than five servings of vegetables a week; only 22.4% of women and 17.7% of men consumed less than two servings a week, and 33.8% of men and 27.5% of women reported consuming 2–5 servings per week. 

More than half of the surveyed population (women 66.3% and men 70.1%) reported consuming three pieces/week and fewer than two pieces/day. Only 14.9% of women and 11.3% of men consumed more than three pieces/day of fruit. 

Only 8.8% of the study population consumed more than 200 g of fish per week, and men consumed it more often (9.5%) than women (8.6%). About half of the study population (47.3%) consumed less than 200 g of fish per week, and men consumed it more often (50.7%) than women (46.2%).

Few participants did not use spreadable fat (17.6%) or cooking fat (7.9%). Men were more likely to give up using fat for spreading (16.0%) and cooking fat (7.4%) compared to women (18.2% and 16.0%). Both sexes used vegetable margarine and oil, among others, for cooking (women 57.7% and men 54.5%). Spreadable fats such as lard, blended spread, and butter were used by men (78.4%) and women (73.7%) with the same frequency, with no statistically significant differences between the two (*p* > 0.05) (Table 2).

Table 3 shows the participants’ Nutrition Quality Score. Only 2.4% of the study population followed healthy eating habits. The majority of respondents (57.6%) had average dietary habits, while 40% had unhealthy eating habits.

No significant difference was found between women (2.3%) and men (3.0%) with regard to healthy eating habits (*p* > 0.05) or between men (42.0%) and women (39.2%) using unhealthy eating habits. No significant difference was found between women (58.5%) and men (55.0%) with average dietary habits (*p* > 0.05).

There were no statistically significant differences regarding unhealthy dietary, healthy dietary, and average dietary habits among marital status (*p* > 0.05) and age groups (*p* > 0.05).

Healthy eating habits were most often demonstrated by people with medium/secondary education (2.5%) (*p* > 0.05). Very few people with primary education (7.7%), post-secondary vocational (1.3%), and higher education (2.1%) had healthy eating habits (*p* > 0.05). Unhealthy eating habits were most common among people with secondary education (42.8%) and least likely among people with primary education (30.8%) (*p* < 0.05). Average dietary habits dominated in all research groups, regardless of the level of education (*p* < 0.05). Unhealthy eating habits were most common among professionally active people (35.7%) and least likely among unemployed people (42.2%) (*p* < 0.01). Similarly, average dietary habits dominated among professionally active people (61.8%) and least frequently among unemployed people (55.6%) (*p* < 0.05).

Table 4 shows the results of the logistic regression analysis for unhealthy dietary habits with socio-demographic characteristics, with an OR and 95% CI indicated for unhealthy eating habits. It can be seen that men had a higher risk of unhealthy eating habits compared to women (OR = 1.12; 95% CI: 0.83–1.52; *p* > 0.05). The OR and 95% CI for unhealthy eating habits showed that educational level played a significant role.

A higher level of education was associated with a greater risk of unhealthy eating habits. The univariable logistic regression found that primary care patients with medium/secondary education had a 1.5 times greater risk of unhealthy eating habits, and those with post-secondary vocational education had a 1.75 times greater risk of unhealthy eating habits than those with higher education: for medium/secondary education, OR = 1.46; 95% CI: 1.08–1.97 (*p* < 0.01); for post-secondary vocational education, OR = 1.75; 95% CI: 1.04–2.94 (*p* < 0.05).

In addition, a significant relationship was found between body mass index and unhealthy eating habits. Obese participants had a much higher chance of unhealthy dietary habits than the subjects with a normal BMI (body mass index) (for ≥30 kg/m^2^ BMI OR = 1.46; 95% CI: 1.04–2.07; *p* < 0.05). The multivariable logistic regression showed that only the level of education had a significant impact on dietary habits: for medium/secondary education, OR = 1.40; 95% CI: 1.03–1.91 (*p* < 0.01); for post-secondary vocational education, OR = 1.69; 95% CI: 1.0–2.85 (*p* < 0.05).

It was found that with the increase in unhealthy eating habits within each variable defining habits, there is a noticeable increase in the odds ratio (OR) of BMI groups, but the observed regularity is not statistically significant (*p* > 0.05). The results are presented in Table 5.

A multivariable logistic regression model predicting belonging to the presenting group with unhealthy dietary habits based on socio-demographic characteristics revealed a Cox and Snell R2 goodness-of-fit index of 0.0034 and a Nagelkerke R2 index of 0.047. No statistically significant relationships were observed between unhealthy eating behavior and socio-demographic characteristics in the regression model.

## 4. Discussion

The present study examines the prevalence of eating behaviors among adult primary care patients with the aim of identifying the factors that determine an unhealthy diet.

The results showed that only a small percentage of primary care patients had healthy eating habits (2.4%), with most subjects having average dietary habits (57.6%). Similar results were achieved in a previous study conducted in Poland among the beneficiaries of social welfare [49]. It has also been found that the prevalence of healthy eating in the general population of Poland is six times higher (15%) than among current primary care patients [50].

In the present study, 40% of respondents reported consuming a low-quality diet. These results are better than those of previous studies conducted in Poland, where 60% of respondents reported consuming a low-quality diet and 90.7% of the disadvantaged population [49,50].

This study used a simple tool, the DQS (Dietary Quality Score), to determine whether a given population is consuming a healthy diet. The DQS score also indicates which element of the diet is being consumed at a low, insufficient, or adequate level. DQS not only determines the dietary behavior of a given population but also allows for the development of targeted interventions to improve behavior regarding those food components that are not being consumed sufficiently [49].

In our study, men ate more fruit per day or week than women. Different results were obtained in Poland among populations with low socio-economic status, where women ate fruit more often during the day or week than men [49].

Certain social patterns have been demonstrated in the motivation to eat. It has been found that men with a low level of education and low income eat vegetables and fruit less often [51,52]; indeed, other studies also confirm that people with the lowest education tend to choose fruit and fish less often [53]. This is also confirmed by our study results, where the majority of surveyed men had secondary or higher education.

The results of our study indicate that the recommended doses of vegetables and fruit were consumed by 49.7% and 13.9% of respondents, respectively. Lower results were obtained in a study of populations with low socio-economic status, where only 11.6% consumed sufficient vegetables and 12.3% [49]. Earlier studies found that 44–80% of respondents consume fruit and vegetables in recommended doses in the diet of Poles [50,54]. A survey of university students found that over half did not eat fruit, and 40% did not eat vegetables at least once a day [55].

Other studies indicate that the mean daily intake of fruit and vegetables varies between countries; e.g., higher consumption of vegetables and fruit was found in Italy (239 and 199 g/day, respectively) and lower consumption in the Czech Republic (95 and 118 g/day). Denmark and the Czech Republic also observed higher vegetable consumption among people with higher education, while Italy and France observed similar vegetable consumption across educational levels, consistent with previous studies in European populations [56]. A survey conducted among primary care patients in Ryiadh showed that 48.4–52.0% of participants consumed fruit and vegetables fewer than three times a week [57].

It is recommended to eat more than 200 g of fish per week. In our population of primary care patients, neither men nor women consumed the recommended amount of fish. This portion of fish per week reduces the risk of death due to coronary heart disease, cardiovascular disease, and total mortality [58]. 

The women ate fish much less often. Previous studies in Poland confirmed low fish consumption among populations with low socio-economic status [49] and that only 52.4% of university students in Poland ate fish several times per month [55]. Higher fish consumption is generally observed among women and people with higher education, which may be due to health reasons [59]. Studies conducted in European countries indicate higher mean fish consumption in France (34.4 g/day) and Italy (44.6 g/day) [56]. Prospective studies have shown a beneficial effect of frequent fish consumption on the risk of cardiovascular diseases, which is attributed to the n-3 fatty acids contained in fish, mainly eicosapentaenoic acids (EPA) and docosahexaenoic acids (DHA) [60].

The type of cooking fat is an important component of an unhealthy diet. Our respondents also too often chose the wrong type, as noted previously in a study on a population with low economic status in Poland [49]. Other studies indicate that high socioeconomic status and health awareness are strongly associated with choosing to use ‘healthy’ unsaturated oil compared to palm oil, which is consistently used by people of the lowest socioeconomic status [61]. In our study, the consumption of spreadable fats (lard, butter, and spreads) was almost ten times higher than that of vegetable margarine. This tendency was observed among both men and women. Similarly, previous research indicates that consumption of spreadable fats was twice as high as that of vegetable margarine among people with low socio-economic status [49]. Research indicates that unhealthy dietary behaviors, such as more frequent consumption of SFA (saturated fat) fats instead of PUFA (polyunsaturated fat), are associated with a high risk of developing prostate cancer in men and breast cancer in women [62,63]. A low omega-3/omega-6 ratio in the diet and being overweight may increase the risk of breast cancer [63]. Increased consumption of omega-3 fatty acids from plants and fish, and eating a diet rich in vegetables, fruits, whole grains, and nuts are effective in preventing coronary heart disease [64]. A study in Hungary among primary care patients showed that in both sexes, fats accounted for a higher (39%) share of energy intake than recommended [65]. 

Our statistical analysis of socio-demographic data, health status, and diet used by respondents showed that the level of education influenced eating behavior. Respondents with secondary and post-secondary vocational education were more likely to present unhealthy eating behaviors than people with higher education. This is confirmed by research conducted in European countries and the USA, where the level of education was most strongly associated with a healthy diet [66,67,68,69]. 

In the present study, people with obesity had a higher risk of developing unhealthy eating habits compared to people with a normal weight; however, this was not confirmed in multivariate logistic regression. The remaining factors, viz. age, sex, marital status, professional situation, and the number of chronic diseases, did not have any significant influence on the choice of diet quality.

All adults should eat a healthy diet rich in whole grains, vegetables, fruit, fish, nuts, and lean plant or animal protein. For overweight and obese adults, caloric restriction and counseling to maintain a healthy weight are recommended [70].

To maintain healthy eating habits, more sources of information about healthy eating need to be provided. It should be remembered that unhealthy eating behaviors from childhood continue into adulthood, and it is important to begin health education as early as possible [71,72]. Introducing interventions at a younger age improves eating behaviors, particularly regarding increased fruit and vegetable consumption and fewer sweetened drinks, reducing the risk of obesity in the future [73]. However, raising awareness about healthy eating habits is possible even at an older age [67]. Studies have shown that educational programs can influence eating habits and reduce the level of cholesterol and saturated fats in the diet among people with both high and low socio-economic status [74].

A brief educational intervention by a primary care physician can induce dietary changes that may lower BMI and reduce the risk of chronic disease in generally healthy adults [75]. Such lifestyle recommendations are highly valued by patients but are still underutilized because they are mainly given in the context of disease [76].

Nutritional intervention programs should aim to increase knowledge of what makes a healthy diet and awareness of the health benefits of adopting it. A good environment for these interventions is primary health care services and nutritional counseling provided by general practitioners.

Developing a better understanding of behaviors that promote healthy eating, education, raising awareness, and the impact of healthy eating habits on health among patients using primary health care should be a priority in public health. Diet is a modifiable factor in maintaining health and reducing the risk of chronic disease. Better education and health promotion will increase the chance of achieving satisfactory results among primary health care patients. It is worth taking into account teamwork, which is the highest-ranked domain of work in a medical office in Poland [77].

Strengths of the study. This is the first study on dietary behaviors among primary healthcare patients in the urban population of Poland. It also examines the prevalence of healthy eating behaviors among respondents and records certain determinants of diet quality. The analysis was based on the use of DQS, a widely used tool, making it possible to assess the quality of the diet of the subjects. The results provide the basis for developing programs for primary care patients rather than the population in general, thus allowing them to achieve better results. The study described an urban population, ensuring the generalizability of the results to other populations and other urban areas.

Limitations of the study. This study is a cross-sectional study conducted at a single time point, focusing on the current situation of the studied patients. The lack of statistically significant results between the BMI groups and dietary habits may be due to the small study group. In the analyzed group, the developed multivariable logistic regression model had low prognostic ability based on socio-demographic characteristics. No statistically significant relationships were observed between unhealthy eating behavior and socio-demographic characteristics. Using different values as references for ordinal variables could slightly improve the results. However, the obtained results indicate that unhealthy eating behaviors are complex in nature and cannot be explained solely on the basis of socio-demographic characteristics.

Furthermore, no information was provided regarding previous healthy or unhealthy eating habits, e.g., whether it was the influence of family, school, household, or family income. Changing unhealthy eating habits is a long-term process that needs to be repeated and verified. Healthcare professionals, i.e., doctors and nurses, as well as public health educators, can play a huge role by motivating patients to change, adopt, and maintain good eating habits. 

## 5. Conclusions

The incidence of unhealthy eating behaviors among primary care patients in Poland is much lower than in the general population. The level of education is significantly correlated with the prevalence of unhealthy eating behaviors in the studied population. This factor should be considered in the promotion of healthy eating behaviors and nutritional counseling interventions conducted by family physicians in primary health care. High diet quality is the unifying element of all dietary recommendations and should be the main focus of health promotion and national food policy. Further research is needed to elucidate the causal relationship between dietary patterns, health management, and chronic disease.

## Figures and Tables

**Table 1:** The socio-demographic characteristics of the study population (*n* = 896).

Variable	Total *n* = 896 (%)	Women *n* = 665 (74.2%)	Men *n* = 231 (25.8%)	*p*-Value
Age (years)				
<30	256 (28.6)	226 (88.3)	30 (11.7)	
30–39	123 (13.7)	105 (85.4)	18 (14.6)	*p* < 0.001
40–49	215 (24.0)	166 (77.2)	49 (22.8)	
50–59	105 (11.7)	68 (64.8)	37 (35.2)	
60+	197 (22.0)	100 (50.8)	97 (49.2)	
Education				
Primary	26 (2.9)	13 (50.0)	13 (50.0)	
Medium/Secondary	510 (56.9)	361 (70.8)	149 (29.2)	*p* < 0.001
Post-secondary vocational	74 (8.3)	57 (77.0)	17 (23.0)	
Higher	286 (31.9)	234 (81.8)	52 (18.2)	
Marital status				
Single	394 (44.0)	329 (83.5)	65 (16.5)	
Married	374 (41.7)	251 (67.1)	123 (32.9)	*p* < 0.001
Widowed	69 (7.7)	41 (59.4)	28 (40.6)	
Divorced	59 (6.6)	44 (74.6)	15 (25.4)	
Professional situation				
Unemployed	45 (5.0)	39 (86.7)	6 (13.3)	
Professionally active	552 (61.6)	418 (75.7)	134 (24.3)	*p* < 0.001
Pensioner	144 (16.1)	66 (45.8)	78 (54.2)	
Student/pupil	155 (17.3)	142 (91.6)	13 (8.4)	
Chronic diseases (number)				
0	420 (46.9)	342 (81.4)	78 (18.6)	
1	205 (22.9)	158 (77.1)	47 (22.9)	*p* < 0.001
2	109 (12.2)	78 (71.6)	31 (28.4)	
≥3	162 (18.0)	87 (53.7)	75 (46.3)	
Body mass index				
normal (<25 kg/m^2^)	494 (55.1)	441 (89.3)	53 (10.7)	
overweight (≥25–<30 kg/m^2^)	227 (25.4)	143 (63.0)	84 (37.0)	*p* < 0.001
obesity (≥30 kg/m^2^)	175 (19.5)	81 (46.3)	94 (53.7)	

**Table 2:** Frequency of consumption of fruit, vegetables, fish, and fats among primary health care patients (N = 896).

Food	Frequency of Consumption	Total *n* = 896 (%)	Women *n* = 665 (%)	Men *n* = 231 (%)	*p*-Value
Vegetables	<2 servings/week	190 (21.2)	149 (22.4)	41 (17.7)	0.2121
	2–5 servings/week	261 (29.1)	183 (27.5)	78 (33.8)	0.2913
	>5 servings/week	445 (49.7)	333 (50.1)	112 (48.5)	0.4967
Fruit	<3 pieces/week	168 (18.8)	125 (18.8)	43 (18.6)	0.1875
	3 pieces/week and <2 pieces/day	603 (67.3)	441 (66.3)	162 (70.1)	0.6730
	>3 pieces/day	125 (13.9)	99 (14.9)	26 (11.3)	0.1395
Fish	<200 g/week	424 (47.3)	307 (46.2)	117 (50.7)	0.4732
	>200 g/week	79 (8.8)	57 (8.6)	22 (9.5)	0.0881
	No intake	393 (43.9)	301 (45.2)	92 (39.8)	0.4386
Fat (spreadable)	None	158 (17.6)	121 (18.2)	37 (16.0)	0.1763
	vegetable margarine, margarine,	67 (7.5)	54 (8.1)	13 (5.6)	0.0748
	blended spread, butter, lard	671 (74.9)	490 (73.7)	181 (78.4)	0.7489
Fat (cooking)	olive oil/none	71 (7.9)	54 (8.1)	17 (7.4)	0.0792
	oil, vegetable margarine	510 (56.9)	384 (57.7)	126 (54.5)	0.5692
	butter, margarine, lard, blended, spread	315 (35.2)	227 (34.2)	88 (38.1)	0.3516

**Table 3:** Categories of DQS and socio-demographic characteristics of the study population.

Variable	Total	Dietary Habits					
		Unhealthy		Average		Healthy	
	*n* = 896 (%)	*n* = 358 (40.0%)	*p*-Value	*n* = 516 (57.6%)	*p*-Value	*n* = 22 (2.4%)	*p*-Value
Sex							
Men	231 (25.8)	97 (42.0)	*p* > 0.05	127 (55.0)	*p* > 0.05	7 (3.0)	*p* > 0.05
Women	665 (74.2)	261 (39.2)		389 (58.5)		15 (2.3)	
Age (years)							
<30	256 (28.6)	102 (39.8)	*p* > 0.05	146 (57.0)	*p* > 0.05	8 (3.1)	*p* > 0.05
30–39	123 (13.7)	44 (35.8)		79 (64.2)		- (0.0)	
40–49	215 (24.0)	81 (37.7)		127 (59.1)		7 (3.2)	
50–59	105 (11.7)	43 (41.0)		61 (58.1)		1 (0.9)	
60+	197 (22.0)	88 (44.7)		103 (52.3)		6 (3.0)	
Education							
Primary	26 (2.9)	8 (30.8)	*p* < 0.05	16 (61.5)	*p* < 0.05	2 (7.7)	*p* > 0.05
Medium/Secondary	510 (56.9)	218 (42.8)		279 (54.7)		13 (2.5)	
Post-secondary vocational	74 (8.3)	35 (47.3)		38 (51.4)		1 (1.3)	
Higher	286 (31.9)	97 (33.9)		183 (64.0)		6 (2.1)	
Marital status							
Single	394 (44.0)	162 (41.1)	*p* > 0.05	221 (56.1)	*p* > 0.05	11 (2.8)	*p* > 0.05
Married	374 (41.7)	154 (41.2)		213 (56.9)		7 (1.9)	
Widowed	69 (7.7)	24 (34.8)		44 (63.8)		1 (1.4)	
Divorced	59 (6.6)	18 (30.5)		38 (64.4)		3 (5.1)	
Professional situation							
Unemployed	45 (5.0)	19 (42.2)	*p* < 0,01	25 (55.6)	*p* < 0,05	1 (2.2)	*p* > 0.05
Professionally active	552 (61.6)	197 (35.7)		341 (61.8)		14 (2.5)	
Pensioner	144 (16.1)	66 (45.8)		75 (52.1)		3 (2.1)	
Student/pupil	155 (17.3)	76 (49.0)		75 (48.4)		4 (2.6)	
Chronic diseases (number)							
0	420 (46.9)	159 (37.9)	*p* > 0.05	252 (60.0)	*p* < 0,05	9 (2.1)	*p* > 0.05
1	205 (22.9)	87 (42.4)		113 (55.1)		5 (2.5)	
2	109 (12.2)	39 (35.8)		67 (61.5)		3 (2.7)	
≥3	162 (18.0)	73 (45.1)		84 (51.9)		5 (3.0)	
Body mass index							
normal	494 (55.1)	191 (38.7)	*p* < 0.05	292 (59.1)	*p* < 0,05	11 (2.2)	*p* > 0.05
overweight	227 (25.4)	83 (36.6)		139 (61.2)		5 (2.2)	
obesity	175 (19.5)	84 (48.0)		85 (48.6)		6 (3.4)	

**Table 4:** The odds ratio of unhealthy dietary habits by socio-demographic characteristics.

Variable	Total	Unhealthy Dietary Habits		Univariable		Multivariable	
				Logistic Regression			
	*n* = 896	*n* = 358	*p*-Value	Odds Ratio (OR)	Confidence Intervals (95% CI)	Odds Ratio (OR)	Confidence Intervals (95% CI)
Sex							
Men	231 (25.8)	97 (42.0)	0.4634	1.12	(0.83–1.52)		
Women	665 (74.2)	261 (39.2)		1.00	Ref.		
Age (years)							
<30	256 (28.6)	102 (39.8)	0.5195	1.00	Ref.		
30–39	123 (13.7)	44 (35.8)		0.84	(0.54–1.31)		
40–49	215 (24.0)	81 (37.7)		0.91	(0.63–1.32)		
50–59	105 (11.7)	43 (41.0)		1.05	(0.66–1.66)		
60+	197 (22.0)	88 (44.7)		1.22	(0.84–1.78)		
Education							
Primary	26 (2.9)	8 (30.8)	0.0354	0.87	(0.36–2.06)	0.62	(0.33–1.94)
Medium/Secondary	510 (56.9)	218 (42.8)		1.46	(1.08–1.97) **	1.40	(1.03–1.91) *
Post-secondary vocational	74 (8.3)	35 (47.3)		1.75	(1.04–2.94) *	1.69	(1.0–2.85) *
Higher	286 (31.9)	97 (33.9)		1.00	Ref.	1.00	Ref.
Marital status							
Single	394 (44.0)	162 (41.1)	0.3315	1.59	(0.88–2.87)		
Married	374 (41.7)	154 (41.2)		1.59	(0.88–2.88)		
Widowed	69 (7.7)	24 (34.8)		1.22	(0.58–2.56)		
Divorced	59 (6.6)	18 (30.5)		1.00	Ref.		
Professional situation							
Unemployed	45 (5.0)	19 (42.2)	0.0085	1.00	Ref.		
Professionally active	552 (61.6)	197 (35.7)		0.76	(0.41–1.41)		
Pensioner	144 (16.1)	66 (45.8)		1.16	(0.59–2.28)		
Student/pupil	155 (17.3)	76 (49.0)		1.32	(0.67–2.57)		
Chronic diseases (number)							
0	420 (46.9)	159 (37.9)	0.0684	1.00	Ref.		
1	205 (22.9)	87 (42.4)		1.21	(0.86–1.70)		
2	109 (12.2)	39 (35.8)		0.91	(0.59–1.42)		
≥3	162 (18.0)	73 (45.1)		1.35	(0.93–1.94)		
Body mass index							
normal	494 (55.1)	191 (38.7)	0.0461	1.00	Ref.	1.00	Ref.
overweight	227 (25.4)	83 (36.6)		0.91	(0.66–1.27)	0.88	(0.63–1.23)
obesity	175 (19.5)	84 (48.0)		1.46	(1.04–2.07) *	1.38	(0.97–1.98)

*p* < 0.05 *; *p* < 0.01 **; Ref.—Reference; fully adjusted model, including all statistically significant data.

**Table 5:** Relationship between BMI groups and eating habits (ordinal logistic regression).

Food	Frequency of Consumption	Total *n* = 896 (%)	OR	95%CI	*p*-Value
Vegetables	<2 servings/week	190 (21.2)	1.19	0.79–1.55	0.5675
	2–5 servings/week	261 (29.1)	1.20	0.67–1.39	0.8340
	>5 servings/week	445 (49.7)	1.00	Ref.	Ref.
Fruit	<3 pieces/week	168 (18.8)	1.27	0.77–1.98	0.3833
	3 pieces/week and <2 pieces/day	603 (67.3)	1.19	0.61–1.20	0.3518
	>3 pieces/day	125 (13.9)	1.00	Ref.	Ref.
Fish	<200 g/week	424 (47.3)	1.15	0.62–1.06	0.1223
	>200 g/week	79 (8.8)	1.27	0.58–1.51	0.7960
	No intake	393 (43.9)	1.00	Ref.	Ref.
Fat (spreadable)	None	158 (17.6)	1.19	0.72–1.44	0.9179
	vegetable margarine, margarine,	67 (7.5)	1.29	0.73–1.97	0.4853
	blended spread, butter, lard	671 (74.9)	1.00	Ref.	Ref.
Fat (cooking)	olive oil/none	71 (7.9)	1.30	0.54–1.49	0.6785
	oil, vegetable margarine	510 (56.9)	1.15	0.83–1.44	0.5461
	butter, margarine, lard, blended, spread	315 (35.2)	1.00	Ref.	Ref.

Ref.—Reference.

## Data Availability

The datasets used and/or analyzed during the current study are available from the corresponding author upon reasonable request due to privacy reasons.

## Supplementary Materials

The following supporting information can be downloaded at: https://www.mdpi.com/article/10.3390/nu16070925/s1, Table S1: Characteristics of the DQS (Dietary Quality Score); Table S2: DQS categories.
