# Supplementary material for: Dietary Behavior and Determinants of Diet Quality among Primary Health Care Patients in Poland
Source: Nutrients. 2024 Mar 23;16(7):925. doi: 10.3390/nu16070925 (PMC11013156; doi:10.3390/nu16070925)
Supplement: Supplementary file 1 [file nutrients-16-00925-s001.zip › nutrients-2923157-supplementary.pdf]

Supplementary materials

Table S1. Characteristics of DQS (Dietary Quality Score).

Food	Frequency	Score
Vegetables	>5 servings/week<br>2-5 servings/week<br><2 servings/week	2 points<br>1 point<br>0 point
Fruit	>3 pieces/day<br>>3 pieces/week and <2 pieces/day<br><3 pieces/week	2 points<br>1 point<br>0 point
Fish	>200 g/week<br><200 g/week<br>No intake	2 points<br>1 point<br>0 point
Fat	None	2 points
Fat, spread	Vegetable margarine<br>Butter, blended spread, lard	1 point<br>0 point
Fat, cooking	None/olive oil<br>Vegetable margarine, oil<br>Margarine/butter/blended spread/ lard	2 points<br>1 point<br>0 point
Fat, summarized	6 points, summarized<br>3-5 points, summarized<br>2 points, summarized	2 points<br>1 point<br>0 point

Table S2. DQS categories

Category	Score
Unhealthy dietary habits	0-3 points
Average dietary habits	4-6 points
Healthy dietary habits	7-8 points
